# *“I think it is our responsibility, but not solely our responsibility”*: A qualitative study exploring teachers’ perspectives on promoting mental health in Northwest London primary schools

**DOI:** 10.1371/journal.pone.0336946

**Published:** 2025-12-11

**Authors:** Cecilia N. Ashu, Dougal S. Hargreaves, Tamsin Robinson, Dasha Nicholls, Bina Ram

**Affiliations:** 1 School of Public Health, Imperial College London, London, United Kingdom; 2 NHS Northwest London Integrated Care Board, London, United Kingdom; 3 Department of Brain Sciences, Imperial College London, London, United Kingdom; Universiti Sains Malaysia, MALAYSIA

## Abstract

In England, 1 in 5 children and young people (CYP; aged 8–25 years) has a probable mental disorder with higher rates among those living in poverty, and among white children compared with other ethnic groups. However, in the UK, research shows that the prevalence of mental health conditions and associated service use differs among some ethnic minority groups (e.g., Asians) suggesting potential unmet mental health need. Early interventions have been shown to improve life outcomes, and UK government policies encourage the promotion of mental health and wellbeing in schools and colleges, but poor mental health continues to rise. Despite evidence showing that mental health problems occur as early as age 5 years, limited research focusses on primary schools (children aged 5–10 years). Northwest London (NWL), UK, is a diverse region in London, is in the top 20% most deprived, and has a high demand for CYP mental health services. The aim of our study was to explore teachers’ perspectives of promoting positive mental health in NWL primary schools. We created a semi-structured interview based on policy guidance. Nine teachers were recruited and interviewed during June and July 2024. Thematic analysis identified six overarching themes: (1) mental health needs; (2) responsibility; (3). factors contributing to poor mental health; (4). barriers and (5) facilitators to providing support; and (6) collaboration. Subthemes included lack of skills to address the broad spectrum of mental health needs, funding, resources, and lack of support systems to ensure all children receive the right support at the right time. Our study highlights implementation gaps for promoting mental health policy in diverse real-world settings, and suggests that whilst schools play an important role for early mental health intervention, wider complexities limit sufficient support provision. Our findings have potential implications for mental health promotion policies in school settings.

## Introduction

The prevalence of mental disorders among children and young people (CYP) aged 8–25 years in England has increased since 2017 [1]. Mental health among CYP has worsened [2] and has increased the demands for mental health services which has been further highlighted by Lord Darzi’s report published in 2024 [3]. The latest data (2023) show that 1 in 5 CYP had a probable mental disorder, an increase from 1 in 8 in 2017 [1]. This is reported to be around 1 in 10 children in primary schools (aged 5–10 years) and 1 in 7 in secondary schools (aged 11–16 years) [4]. More than half of CYP diagnosed with a mental disorder report not having appropriate support at an early age [5]. Evidence shows that experiencing mental health problems prior to the age of 14 years increases the risk of mental disorder persisting into adulthood [6]. Additionally, disparities in mental health continue to widen; poorer mental health rates are higher among those living in poverty, some ethnic minority groups, and those with additional needs and disabilities [7,8] who may also experience inequitable access to mental health services [9,10]. Early intervention is crucial; mental health problems contribute to school absence, potentially impacting education outcomes and employment prospects in later life [1]. Efforts targeted at children in the early years may help reduce mental health need and contribute towards reducing mental health disparities over the life course [6].

Schools are recognised as playing a key role for promoting positive mental health [11,12]. They are included in the THRIVE Framework, an integrated (health, education, social care, and community settings), person-centered, and needs-led system change approach to deliver mental health services to CYP. As part of the framework, i-THRIVE is the implementation of THRIVE in practice to deliver a population health model for CYP mental health, and can refer children to specialist services such as CAMHS (Child and Adolescent Mental Health Services) [13]. In England, education is compulsory for all children from the ages of 5–16 years [14]. Schools are considered ideal public health settings for early interventions to promote positive mental health [15] as most children attend a formal educational establishment and mental health inequalities can be addressed.

In 2018, the Department for Education (DfE) published a review of the content of schools’ policies and practices for promoting mental health [11]. The review, based on 100 schools in England (45 secondary, 45 primary, and 10 special schools), relied on information published on school websites. The ‘desk-based approach’ highlights a research gap of promoting mental health in real-world settings. In 2021, Public Health England published a policy framework outlining eight principles of a whole school or college approach to promoting mental health [16]. These principles require effective leadership and management which supports efforts to promote mental health and wellbeing. DfE argue that the principles will help protect and promote CYP mental health and wellbeing when applied consistently and comprehensively [17,18]. In addition, since 2018/2019, NHS England (NHSE) regional teams have been implementing an initiative to provide Mental Health Support Teams (MHSTs) for schools [19]. MHSTs were initially focused in local areas where there are higher levels of need, inequality and disadvantage [18], led by Integrated Care Systems (ICSs) and Integrated Care Boards (ICBs). As of Spring 2023, MHSTs were reported to be reaching 6,800 schools and colleges and 35% of children in England [18]; the aim is for universal coverage to increase service accessibility and uptake. However, an early evaluation found gaps in MHST service provision in schools including some groups feeling underserved (e.g., those with Special Educational Needs and Disabilities (SEND), those from ethnic minority backgrounds, and children with challenging family or social circumstances) [20].

Despite indications of mental disorders being present as early as aged 5 years [21] and early interventions in school settings being widely recognised as important, qualitative evidence on the implementation of the promotion of mental health in primary schools is an under-researched area [22]. Previous studies report teachers’ perceptions using quantitative methods [23–25] or desk based research [11] which lack opportunities for an in-depth understanding around the complexities of implementing mental health promotion in schools. Furthermore, studies more often focus on adolescents (aged 11 + years) in secondary schools, which show that teachers’ are aware of the importance of supporting CYP mental health but lack the training and skills needed to provide adequate support [23,26–29]. Although views of secondary school teachers may corroborate with primary school teachers, their roles are fundamentally different; primary school teachers spend more time with children as they teach year-group cohorts and are potentially better placed to identify early developmental and behavioural characteristics of children’s mental well-being, whilst secondary school teachers teach subject-specific cohorts [30]. Additionally, few UK qualitative studies use in-depth qualitative methods with primary school teachers, and any relevant studies were published ≥5 years ago [31–33]. Mental health problems among CYP have worsened due to the Covid-19 pandemic (e.g., social isolation, anxiety), which may have increased challenges for teachers. Exploring in-depth views of primary school teachers is important to understand implementation of mental health policy in practice, which will inform intervention strategies to improve CYP mental health.

North West London (NWL), London, UK, has high levels of CYP unmet mental health need and is in the 20% most deprived areas of the national population [7]. NWL is diverse; it includes those from ethnic minority communities, people with SEND, and those with multi-morbidities. Improving mental health is one of five key priorities of the NWL ICB and Integrated Care Partnerships to improve access rates to CYP mental health services [34]. Whole school approaches for good mental health can play a crucial role in supporting CYP who may not yet meet thresholds for service support, and for those on waiting lists for mental health services. Therefore, understanding the implementation of mental health practices and policies in schools using qualitative approaches is important to complement quantitative methods. The aim of our study was to gain a better understanding of the implementation of mental health policies in real-world settings, by exploring teachers’ perspectives of mental health promotion in primary schools.

## Materials and methods

### Study design

This is a qualitative study exploring implementation on the promotion of mental health policy in primary schools by using semi-structured interviews to identify teachers’ perspectives and experiences.

### Ethics

Review for ethical approval was not required as the study’s focus was to evaluate a policy; confirmation was received by Imperial College Research Ethics Committee. We completed Imperial College London’s Data Activity Risk assessment Tool (DART) to ensure our data complied with General Data Protection Regulation (GDPR) and data management principles.

All participating teachers were provided with an information sheet about the study, and completion of an online consent form was required before interviews took place. Participation was voluntary and teachers were free to stop the interview and/or withdraw at any time. All data were pseudo-anonymised, password protected and saved on a secure server at Imperial College London which are only accessible to the study team.

### Policy for promoting a whole school approach to mental health and wellbeing

**Fig 1:**
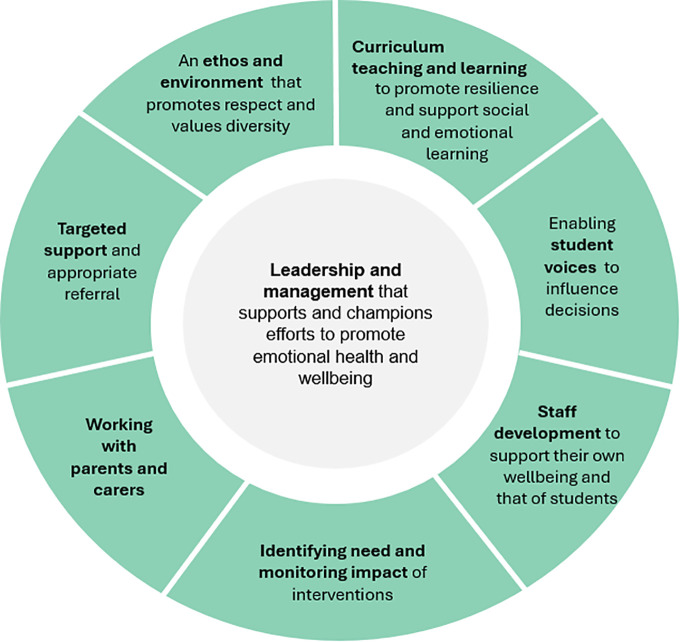
Eight principles for a whole-school approach to promoting mental health and wellbeing.

Government policy for a whole school approach for promotion of mental health (Fig 1) centres around the principle of (1) ‘leadership and management’ which supports efforts to promote emotional health and wellbeing, and is surrounded by seven other principles: (2) to promote resilience and support social and emotional learning though curriculum teaching and learning; (3) enabling student voice to influence decisions; (4) staff development to support their own wellbeing and that of students; (5) identifying need and monitoring impact of interventions; (6) working with parents and carers; and (7) targeted support and appropriate referral; and (8) an ethos and environment that promotes and respects values and diversity [16].

### Semi-structured interview development

Guided by the government principles [16], a review of published government reports and NHS guidance on mental health in schools [7,11,18,35], relevant literature [9,20,24,36], and input from NWL ICB, we developed our semi-structured interview guide to address: (1) how mental health of children is perceived by teachers and their responsibilities; (2) teachers’ direct experiences of children’s mental health needs; and (3) school policies that promote good mental health (S1 Fig).

### Participant recruitment and procedures

At the time of this project (summer 2024), there were 1,828 open state-funded primary schools in all Greater London boroughs; 390 of these were in NWL. We downloaded contact details (head teacher name, school address, school email address) of all NWL these schools which are publicly available from the UK government website [37], and created a school dataset by each NWL borough. We also downloaded data on school characteristics to compare differences between NWL and non-NWL schools [37–39]. We aimed to recruit a minimum of 8 and maximum of 10 NWL primary school teachers, guided by previous research [40] of the likely number needed within time and resource constraints.

We used a purposive and convenience sampling approach and invited a total of 166 primary schools. Initially, 16 schools (x2 schools per borough) were each contacted by email. Emails were addressed to the head teacher at the school but any teacher from the school was eligible to participate but limited to one teacher per school to ensure we captured views of teachers from different schools and boroughs. Due to low response rates, we sent an email to an additional 50 schools two weeks after contact with the first set of schools. This continued to yield few responses. We invited a further 100 schools by email a week later.

Teachers who responded to the invitation were contacted by phone to discuss the project. Upon verbal consent, a convenient date and time was arranged for the interview. Teachers were required to complete an online consent form (created using Qualtrics software) prior to the interview which included agreement for the interview to be recorded, data to be used, confidentiality, General Data Protection Regulations (GDPR), and to provide optional consent for the teacher and/or school to be acknowledged by name in outputs emanating from the project. Interviews took place over a five-week period during June and July 2024 and conducted online using the online platform Microsoft Teams. Interviews lasted an average 33 minutes and all interviews were recorded and transcribed. Transcripts were exported into the qualitative analysis software NVivo [41]. All participating teachers received a £25 incentive shopping voucher.

### Analysis

#### School characteristics.

We compared school characteristics of NWL schools (type, size, sex, ethnic composition, number of children with SEND, number of children with English as a second language, number of children eligible for free school meals, Ofsted rating, and a measure of deprivation using the Lower Super Output Area from the Income Deprivation Affecting Children Index (IDACI)), with non-NWL schools. All data were categorical; chi-squared or Fisher’s exact statistical tests were used to assess differences between NWL and non-NWL schools. Data was analysed using Stata v13.

#### Thematic analysis.

Each transcript was independently read by two researchers (CA and BR). We used thematic analysis to code and extract common themes. These were independently derived by both researchers who met to discuss their codes and agreed the final themes and sub-themes. This method allowed for the validity of categories assigned and to reduce researcher bias [42]. A third researcher (DH) was consulted for any discrepancies or disagreements.

### Public involvement and engagement

Our semi-structured interview was created in consultation with a wider team of academic and non-academic researchers at Imperial College London, the National Institute of Health and Care Research (NIHR) NWL Applied Research Collaboration (ARC), and NWL ICB.

## Results

### School characteristics

Schools differed in characteristics of schools in NWL compared with schools in all other London regions in school type (p = 0.000), number of children (p = 0.02), ethnic composition (p = 0.000), number with English as a second language (p = 0.000), and deprivation (p = 0.000; S1 Table).

### Participants

A total of nine teachers (x4 Special Educational Need Co-ordinators (SENCO), x2 class teachers (Year 2 and Year 6), and x1 each of a year group head, head teacher, and deputy head/mental health lead) participated, yielding a 5.42% response rate. Teachers represented nine different schools from six of the eight NWL boroughs: Brent, Ealing, Hammersmith and Fulham, Harrow, Hillingdon, and Hounslow. Eight of the teachers were female.

### Theme findings

Analysis of the interview transcripts identified six overarching themes: (1) mental health needs; (2) responsibility; (3) factors contributing to poor mental health; (4) barriers to providing support; (5) facilitators to providing support; and (6) collaboration. Some of the overarching themes included sub-themes and are described below. To ensure anonymity of participants, the quotes used from the interviews do not include the corresponding teacher name, role, or the borough in which their school was located.

#### Theme 1: Mental health needs.

Teachers identified a range of children’s mental health needs which included anxiety, wellbeing, emotional and behavioural problems, and mental health literacy. The most common mental health condition was anxiety which teachers described as having many different contributing factors ranging from separation from parents to household factors, from cost-of-living to family issues:

*“We’re seeing a lot of emotional based school anxiety…even at the lowest level, it can just be that separation anxiety from parents…so I would say definitely anxiety and very much school related anxieties”.*

*“…they shouldn’t be worrying about how to provide the food on the table. They shouldn’t be worrying about the mom lost the job. Those kinds of things. They’re worried”.*

*“…we have a lot of children that are experiencing high levels of anxiety and that is presenting itself in many ways in the classroom…. their anxiety in their mental health needs have meant that school is not a safe place, that they don’t even feel comfortable in coming”.*

Within this theme, the sub-theme emotional and behavioural problems (e.g., anger and disruptive behaviour in class, and diagnosed and undiagnosed attention deficit hyperactivity disorder (ADHD)) emerged; this was having an impact on children’s own learning and also leading to disruption to others.

*“when you do see somebody having and displaying very dysregulated behaviour, we then see the impact of that on other children in the class”.*

Mental health needs among some children led to issues around safeguarding; incidents of emotional and behavioural problems raised concerns about a child’s welfare:

“*and it’s been as extreme as a child…saying that when they went home, they wanted to kill themselves*”.

Through this theme, teachers shared the spectrum of different mental health needs they were addressing and highlighted the impact this was having on children’s emotional, and psychological wellbeing.

#### Theme 2: Responsibility.

Teachers shared their views on the increased responsibility on them/their school to manage children’s mental health needs. Most acknowledged that outside of parental/guardian care, they (the teachers) spent the most time with children on a regular basis. This placed responsibility to identify signs of poor mental health and work towards providing support alongside their challenging responsibilities of delivering the curriculum. This additional work was placing a greater need on them beyond their role of being a teacher:

*“…where children are going to be seen on a daily basis like where else is this child who is like 5 years old or six years old, who else is going to be able to pick up on it?”*

*“We’re more than just educators now. Whether that’s been forced on us or whether we’ve, you know, it’s just for the best of the child, but it’s looking at, I suppose, looking at the child holistically”.*

Alongside the increasing responsibility, a subtheme emerged around the burden on staff. The increased responsibility was having an impact on staff mental health where schools did not always have support in place for teachers. A few teachers expressed this greater responsibility had led to teachers leaving the profession or often taking periods of sick leave due to stress.

*“In order to create healthy students and healthy children, and we have to start looking after first the staff”.*

#### Theme 3: Factors affecting poor mental health.

Teachers shared that the mental health needs of children, particularly anxiety, were influenced by factors outside the control of the school.

*“…constant moving and not feeling like you’re at home must have an effect on your mental health”.*

*“… parent has been suffering some mental health issues and… it’s interesting how that impacts on that child”*.

Implications caused by the COVID-19 pandemic on young people’s mental was identified as a subtheme; teachers shared that limited interaction during the pandemic led to negative impacts on children’s social skills, language, and academic achievement. Teachers expressed that during the pandemic, children had spent a lot of time on electronic devices such iPads which impacted their social and interaction skills when they returned to school and in turn, increased their anxiety.

*“So, you do have ‘the tablet family’ where the tablet is everything that the child does”.*

#### Theme 4: barriers to promoting mental health in schools.

Teachers commonly identified financial barriers as contributing to the lack of mental health promotion in schools. Limited funding led to limited resources for teachers, and restricted opportunities to invest in adequate support for children with mental health needs. Financial implications prevented training opportunities for existing staff to support the range of different mental health needs. Teachers shared that they did not have the necessary skills to support the children adequately.

*“…the barrier to us is the funding. We don’t receive funding for this provision. We have to ring fence that, raise funds for it, whatever. But if we don’t address that, where do the children go?”*

*“Don’t think there’s enough training on special needs and mental health when you’re qualifying to be a teacher… we haven’t got budget to train everybody”.*

A subtheme of ‘language’ emerged under the theme of ‘barriers to promoting mental health in schools’ which was identified as challenge when addressing mental health need. NWL has a high diverse population group with one school reporting that 98% of their children had English as a second language. It was therefore challenging for children to express how they were feeling for teachers to be able to help. This was resulting in a significant barrier for teachers to identify mental health issues:

*“They’ve got no English.... How do you know if they’ve got mental health issues, I don’t think you would ever really know….. so I think English is an additional language, could be a very, very, big barrier for children”.*

#### Theme 5: Facilitators to promote mental health in schools.

Alongside barriers, teachers also discussed mental health promotion they were implementing at their schools and the further support they would welcome. Some of the schools had access to Emotional Literacy Support Assistants (ELSAs) who supported children to talk about their emotions, whilst others provided a ‘worry box’ to share their emotions. Other schools had a school counsellor in place, and those that did not have a school counsellor expressed their school would benefit from having this type of support. There was general consensus among teachers that access to counsellors was needed at a greater scale to promote mental health and support to children with mental health need.

*“I feel like there needs to be a ring fenced provision for every school to have a school counsellor, whether that’s half a day, a day. But somebody with that professional expertise and awareness of the bigger view…”.*

While there are many strategies schools can do or are trying to do independently, the value of an external professional may contribute effective impacts to support schools promoting good mental health.

#### Theme 6: Collaboration.

Limited opportunities for working collaboratively with stakeholders including parents and external services were commonly highlighted. Lack of collaboration was viewed by teachers as preventing mental health promotion and to providing adequate support. Teachers felt that despite some teacher and school responsibility for promoting good mental health among children, there needed to be a collective, systems-based approach comprising of family, community (including schools), and health services to better support children with mental health needs. Engaging with parents was very challenging for teachers; when schools tried to engage with parents, there was poor uptake.

*“But then I know as a school, we have tried to engage with parents on mental health and a trauma approach, but the uptake on that has not been the greatest”.*

Engaging with mental health services was also a challenge. There was variation by borough of the reported level of support for primary schools and the ability to access mental health services to refer children. Most teachers who were interviewed had found that children had to be ‘in crisis’ before professional support was offered, and the ‘crisis’ could have been prevented with earlier intervention. Teachers agreed that while referrals were easier when children were in crisis, the long waiting lists and sometimes rejected referrals (due to children not meeting thresholds), resulted in a high level of unmet mental health need.

*“To try and get any professional services to be able to accept children, the threshold to pass to get a referral into CAMHS is so significantly high that a child has to be at crisis point. You know self-harming or have harmed other people”.*

To some teachers, this was felt to be a systemic failure in both education and healthcare systems. They expressed that children are receiving inadequate support when signs of poor mental health are emerging, and difficulties with accessing support services appears to be creating worsened conditions for children in the longer term. Teachers expressed concern of the volume of future treatments needed for children who missed access to early intervention.

*“I’m just like that hurts my heart. I’m like, this is the next generation. These are the children that in a couple of years are going to be looking after me when I’m old and retired. If they don’t have the skills and they’re not resilient and they’re not mentally in a good place. The future looks quite like it looks quite scary”.*

### Implementation of policy recommendations

Though not identified as a theme, a few teachers specifically reported that their school had a mental health policy in place, but these were not necessarily guided by government policies. In practice, implementation of the government principles was challenging. Difficulties with engaging parents/carers and wider networks including community groups and health services resulted in schools’ mental health practices being designed by school staff leaders only, who may not necessarily bring wider perspectives that would benefit effective mental health promotion in schools. Teachers’ views were that effective promotion of children’s mental health and wellbeing required invaluable input from parents/carers who would bring awareness around the complexities of home/family circumstances, whilst health care professionals would provide important contributions of how schools could better work and engage with health services.

## Discussion

The aim of our study was to understand teachers’ perspectives for promotion of mental health policy in primary schools that were located in a diverse region of London with high levels of unmet mental health need among CYP. The themes extracted highlighted the breadth of mental health needs, factors contributing to poor mental health, challenges teachers face in providing adequate support, and the need for collaborative working with families and health services to ensure children receive the right support at the right time. Lack of funding and resources placed immense responsibility on teachers to identify children with mental health needs, although that did not necessarily result in children being provided with adequate support. Concerns were raised about the lack of collaborative working with communities and health services to effectively promote mental health and wellbeing, and teachers not feeling fully equipped with the skills needed to support the range of mental health needs for children who did not always meet the thresholds for support from health services. Our findings highlight the challenges for teachers in the feasibility of implementing mental health policies in real-world settings to support CYP with mental health needs.

The themes extracted from our study demonstrate that the government’s recommended principles for a whole school approach for promoting mental health and wellbeing [16] may be difficult to implement where there are high levels of mental health need, inadequate resources, limited funding, and lack of collaboration. Three of the principles were highlighted throughout our themes which teachers identified as the most challenging to implement: ‘targeted support’, ‘working with parents and carers’, and ‘staff development’. ‘Targeted support’ would require the need for further training for teachers to support children who do not meet referral thresholds. However, further training adds to existing heavy workloads and an additional demand on teachers outside teaching the curriculum. Schools which had ELSAs and school counsellors found that having access to these support provisions were beneficial, but it was not available at all schools due to funding budgets. Increased funding may initiate the implementation of mental health wellness tools such as ELSA which has demonstrated to be effective to improving mental health and wellbeing within schools [43]. Despite there being a widely held view among teachers that counsellors provide expert support for children, there is limited evidence that counselling for this age group is effective. Nonetheless, teachers felt that counsellors take the main responsibility away from class teachers and work well in collaboration with allocated mental health staff (i.e., SENCO leads). Of particular interest was that none of the teachers reported that their school used the provision of MHSTs who are specifically trained to deliver mental health interventions and support teachers [20]. Whether this was because they were unaware of MHST support being available to their schools, or not taking advantage of this service provision needs to be explored further. However, an early evaluation of MHST service delivery reported mixed results; encouraging findings showed that schools reported MHSTs improved staff knowledge and confidence to deal with mental health needs, and CYP accessing MHSTs had positive experiences, but there were challenges with service delivery which was impacted by staff turnover limiting MHST availability, and unclear guidance around school needs and expectations vs the support provision provided by MHSTs [20].

In our study, we found that engaging with families for mental health promotion was challenging for schools despite family and household factors contributing to poor mental health among children. Similar findings were shown in a study conducted in Australia [30]. Another study showed that reluctance of parental engagement could be due to reasons including not wanting to admit there is an issue with their child, fear of being blamed, stigma, and potential cultural barriers [31]. Teachers recognise that good quality school and parent relationships is needed to help the promotion of mental health of children, but it is not straightforward in practice. Furthermore, there was concern among teachers of the impact on staff; the burden on non-trained staff or those who feel they don’t have the required skills to support a range of children’s mental health needs which was affecting their own wellbeing. This finding supports that of other studies which have shown increased levels of stress among teachers based on the responsibility to support CYP with different mental health needs [44,45].

Anxiety was the most common reported mental health problem among children, with several contributing factors identified by teachers including household and family issues, and poverty. Anxiety is associated with children’s poor school attendance, which can impact learning and place them at higher risk of poor academic attainment, social isolation, and unemployment in later life [46]. However, there is a limited high-quality evidence base exploring the relationship between anxiety and school absence despite policy guidance on teachers roles and responsibilities around this issue [47]. Although an association between anxiety and school absence exists, research is needed to understand whether anxiety is on the causal pathway to school absence, or whether school absence increases anxiety preventing children from returning to school.

The whole-school framework promotes a supportive environment and culture which should address mental health needs of all students [16]. The findings from our study suggest some principles of the framework are not easily implemented, particularly around parental engagement and integration between schools and health services which are crucial to support a whole school approach of promoting positive mental health among CYP.

Most studies on promoting mental health in schools are commonly undertaken in secondary schools (CYP aged 11–16 years) which is perhaps due to mental disorders having a peak age of onset around 14 years [2]. However, early intervention which identifies children at risk and ensuring adequate support is provided at an earlier age may reduce worsening of mental health in adolescence. Nevertheless, our findings align with similar studies identifying views of mental health promotion in secondary schools which show that teachers do not feel fully equipped with interpreting signs of mental health problems among CYP [23,26–28], the need more resources to support CYP with their mental health [28,48,49], and the need for more clarity around the division of responsibilities between school staff and health services [48].

Whilst it is recognised that schools play a crucial role in identifying mental health needs, there are potentially greater benefits that could be gained from better promotion of positive mental health in schools [2], increased engagements with parents, better integration of health services with education systems [50] and preventative or early intervention which continues for as long as needed to reduce the risk of worsening mental health over time [2,19,51,52]. However, the pressures placed on teachers and the complexity of their evolving roles as educators requires further investment for policy effectiveness [53].

### Strengths and limitations

To our knowledge, this is the first study to qualitatively assess teachers’ perspectives of the promotion of mental health and wellbeing of children in NWL primary schools. By obtaining views of teachers, our study furthers the understanding of mental health promotion in real-world settings beyond that of ‘desk-based’ and survey evidence. The use of semi-structured interviews allowed participants to express their views openly and provided an in-depth insightful understanding of mental health needs of children, support provision, and implementation of a whole-school approach to address mental health and wellbeing among primary school aged children. Our study included teachers from six (from a possible eight) different NWL boroughs and the inclusion of teachers at different levels, roles, and experience. Our analysis used robust qualitative methods to ensure accuracy which strengthens the reliability of the results. However, our study’s limitations include the small number of teachers which was due to time and resource constraints, therefore a more comprehensive exploration of teachers’ views needs to be explored. In addition, the inclusion of only one teacher from some boroughs may not be representative of all schools within that borough. Our methods of purposive sampling may have resulted in only those teachers who were interested in the topic agreeing to be interviewed. Our study is specific to NWL regions which may not be generalisable to other regions or nationally. Nevertheless, our study has provided important insights from teachers in real-world settings to better understand mental health promotion in primary schools and could be easily scaled up to further investigate feasibility of mental health policies aimed at schools.

For NWL ICB and ICSs, local authorities, schools, and health services, this study offers valuable insights into mental health promotion in primary schools to address mental health needs of CYP and provides the foundation for future research and the potential need for policy development. But complexities around parental engagement and factors outside the schools’ control (e.g., potential home environment stressors) need to be addressed to strengthen collaborations with parents and enhance whole school approaches to improve poor mental health.

Early interventions in primary schools have the potential to reduce risks of worsening mental health over time and reduce mental health disparities for lifelong wellbeing. However, more research is needed on a larger scale and in real-world settings to understand the feasibility of mental health policies aimed at schools which may be contributing to increased pressures on teachers. Future research should also include the views of mental health professionals working within school settings (i.e., MHSTs) and of families.

## Conclusions

Primary schools in the UK are particularly well placed for identifying mental health needs among CYP for preventative and early interventions. However, the promotion of mental health in schools requires further investigation; collaborative approaches between schools, communities, and local health services are needed to reduce unmet mental health need among CYP in NWL.

## Supporting information

S1 Fig Semi-structured interview guide. (PDF)

S1 Table Characteristics of primary schools in Northwest London compared with primary schools in all other London regions. (PDF)
