# Supplementary material for: “I think it is our responsibility, but not solely our responsibility”: A qualitative study exploring teachers’ perspectives on promoting mental health in Northwest London primary schools
Source: PLoS One. 2025 Dec 11;20(12):e0336946. doi: 10.1371/journal.pone.0336946 (PMC12698022; doi:10.1371/journal.pone.0336946)
Supplement: S1 Fig — (PDF) [file pone.0336946.s001.pdf]

S1 Figure. Semi-structured interview guide

Introduction

Hello and thank you for your time today. I am conducting a research project, which, from our invite, you'll know is about teachers' views about schools' mental health policies. We are focusing on schools in North West London. Our overall aim is to gain a better understanding of whether teachers think that the mental health practices and policies in primary schools are effective to support teachers to deal with children's mental health issues and identify what could help improve primary school mental health practices.

All the information that we collect from you will be treated in the strictest of confidence. We will be recording this interview to be able to capture your views accurately. All recordings are used for the research write-up, and we follow strict confidentiality rules as per Imperial's GDPR. We may use quotes from the information that you provide us with today, but neither you nor your school will be identifiable.

The interview should take between 30-45minutes.

Is there anything that you would like to ask me about the project before we start?"

[Start recording]

A few general questions to start off with about you...

1. **What is your role at the school? How long have you been in your role?**

[to the nearest months/years]

[If they have not been in role at the school for very long ask them to draw from knowledge on their current school]

2. **What does your role involve?**

[If they describe something about their role which you don't understand, ask "please can you tell me a bit more about that?"]

[If they are not SENCO lead – 'Do you have a SENCO lead at your school?']

If yes: [If there was a child facing mental health difficulties, would you describe your role as referring straight to the \SENCO lead and if not is something you would do?] - elaborate.

Now a few questions about mental health...

3. **What does mental health for primary school aged children mean to you?**

4. **How much do you think schools are responsible for the mental health of primary school children?**

[Why do you think that is?]

S1 Figure. Continued

5. **Since your role in this school, have you been involved in any way with a child with mental health issues?**

[If no, go to Q6]

[If yes, could you tell me a bit more about that without mentioning any names, and how it was handled?]

[How easy was it to refer?]

[Reiterate confidentiality]

[Were the procedures you followed the usual practice in place within your school?]

6. **How does your school's environment/culture promote good mental health for its pupils? For example, what is currently in place in terms of policies/practices at your school that you are aware of to support mental health of children?**

[Ask for more detail if unclear]

7. **I will now ask you very brief questions: Do your schools mental health policies designed with input from pupils and parents? Yes/No**

8. **On a scale of 1-5 how effective do you think your schools' current policies are effective in supporting children with mental health needs?**

9. **In your experience what are some of the most common mental health issues affecting children in your school?**

[From your perspective do you know why that might be?]

10. **What do you think needs to change or develop to see good mental health; a) practice within your school b) amongst primary school children overall?**

11. **Do you as a teacher feel supported in mental health practices carried out in your school?**

[you mentioned earlier (link back to question 5) that you were involved in dealing with a child with mental health issues – did you feel supported? What would you say your school does to make you feel supported in dealing with children's mental health issues?]

[If they don't feel supported - could you tell me a little more about that]

And finally...

12. **Is there anything else you would like to tell me about that we haven't covered today?**

And that's the end of the interview - thank you very much for your time today. Understanding your views has been extremely helpful for our project. Thanks again for your time.

End recording
