# Supplementary material for: “I think it is our responsibility, but not solely our responsibility”: A qualitative study exploring teachers’ perspectives on promoting mental health in Northwest London primary schools
Source: PLoS One. 2025 Dec 11;20(12):e0336946. doi: 10.1371/journal.pone.0336946 (PMC12698022; doi:10.1371/journal.pone.0336946)
Supplement: S1 Table — (PDF) [file pone.0336946.s002.pdf]

S1 Table. Characteristics of primary schools in Northwest London compared with primary schools in all other London regions*

Schools	NWL (n=390) n (%)	Non-NWL (n=1438) n (%)	All (n=1828) n (%)	pvalue NWL vs non-NWL
**School type**[1]				
Academy / Free	93 (24)	492 (34)	585 (32)	0.000[a]
Local Authority	297 (76)	946 (66)	1243 (68)	
**School size (number of children)**[2]				
Less than 500	290 (75)	1147 (80)	1437 (79)	0.02[a]
500 or more	99 (25)	287 (20)	386 (21)	
**Sex**				
Mixed	389 (100)	1430 (99)	1819 (100)	0.69[b]
Non-mixed	1 (0)	8 (1)	9 (0)	
**Ethnic composition**[3]				
Non-white: Less than 100	174 (45)	792 (55)	966 (53)	0.000[a]
Non-white: 100 or more	216 (55)	646 (45)	862 (47)	
**Number of children with SEND**[4]				
Less than 100	350 (90)	1329 (92)	1679 (92)	0.1[a]
100 or more	40 (10)	109 (8)	149 (8)	
**Children with English as second language**[5]				
Less than 100	71 (18)	467 (33)	538 (30)	0.000[a]
100 or more	315 (82)	955 (67)	1270 (70)	
**Children elgible for free school meals**[2]				
<=100	235 (60)	827 (58)	1062 (58)	0.3[b]
>100	154 (40)	607 (42)	761 (42)	
**Ofsted rating**[6]				
Outstanding/Good	366 (97)	1339 (97)	1705 (97)	0.7[b]
Other	11 (3)	46 (3)	57 (3)	
**LSOA deprivation[7] (quintiles)**				
1 (most deprived)	62 (17)	285 (21)	347 (20)	0.000[a]
2	58 (16)	289 (21)	347 (20)	
3	94 (25)	251 (18)	345 (20)	
4	95 (26)	251 (18)	346 (20)	
5 (least deprived)	62 (17)	284 (21)	346 (20)	

[†]School characteristics for the years 2023/2024; sources: https://explore-education-statistics.service.gov.uk/find-statistics/school-pupils-and-their-characteristics 2023/24; https://explore-education-statistics.service.gov.uk/find-statistics/special-educational-needs-in-england/2023-24; and English Indices of Deprivation 2019 - LSOA Level Income Deprivation Affecting Child Index https://opendatacommunities.org/resource?uri=http%3A%2F%2Fopendatacommunities.org%2Fdata%2Fsocietal-wellbeing%2Fimd2019%2Findices

Percentages may not total 100% due to rounding

[a]Chi-square to test for differences between schools in NWL boroughs and non-NWL boroughs

[b]Fisher's exact to test for differences between schools in NWL boroughs and non-NWL boroughsgroups

[1]School Type: Academy / Free schools include Academy Convertor, Academy Sponsor Led, and Free Schools; Local Authority Schools include Community, Voluntary Aided, and Voluntary Controlled Schools

[2]5 schools with missing data

[3]Non-white includes mixed or mulitole ethnic groups, Asian or Asian British, Black or Black British, and other

[4]SEND - Special Educational Needs and Disabilities; based on number of schools with a designated provision within a mainstream school where pupils with SEN, often with an Education, Health and Care (EHC) plan receive specialised support which can include in separate classes

[5]20 schools with missing data

[6]66 schools with missing data; Other includes 'requires improvement', and 'special measures'

[7]LSOA - Lower Super Output Area; data for 97 schools missing; postcode level deprivation based on IDACI (Income Deprivation Affecting Child Index)
